# Child and Adolescent Psychiatry and Mental Health – development of a new open-access journal

**DOI:** 10.1186/1753-2000-2-22

**Published:** 2008-08-13

**Authors:** Joerg M Fegert, Benedetto Vitiello

**Affiliations:** 1 Department for child and adolescent psychiatry/psychotherapy, University Clinic Ulm, Germany; 2 National Institute of Mental Health, Bethesda, Maryland, USA

## Editorial

It was about one year ago, in June 2007, that the first 4 manuscripts were published in CAPMH, thus marking the birth of a new, open-access, international journal in the field of child and adolescent psychiatry and mental health. Now, at the first birthday, seems the right time for both looking back at the accomplishments of the last year and considering further development of CAPMH.

Since June 2007, we have published 29 peer reviewed articles from all over the world. The average time from initial submission to first editorial decision was 7 weeks, and from initial submission to publication was 5 months. Considering that all submissions undergo rigorous peer-review and that those that are ultimately accepted often had to be revised multiple times, theses figures indicate that CAPMH has been successful in providing researchers with an opportunity to share their work in a timely manner. On a 5-point Likert scale, where 5 indicates maximum satisfaction, the authors' rated the submission process an average of 4.1, the peer review process 4.2, and the production process 4.1. The author overall satisfaction with CAPMH was 4.4. Of the respondents, 86% indicated that they would recommend the journal to a colleague and 100% would publish again in CAPMH.

All manuscripts published in CAPMH are immediately accessible for free in PubMed. In summer 2008 the American Psychological Association has accepted CAPMH in their indexing service PsycINFO. Since the beginning of 2008, CAPMH registered approximately 5,000 accesses per month. Among the highly accessed articles, there were the papers by Basker et al. from India [1], more than 5,000 accesses), by Ginicola from the U.S. [2], more than 2,600 accesses), and by Hammerlynck et al. from the Netherlands [3], more than 2,600 accesses), thus indicating the international scope of CAPMH. Our editorial team is especially committed to providing a broad, worldwide perspective on child and adolescent mental health by encouraging and facilitating submissions from a geographically and culturally diverse pool of contributors. In particular, authors from countries with traditionally limited access to research resources have been encouraged to submit manuscripts and given all possible editorial support throughout the review process. This strategy has allowed the Journal to offer our readers a truly international "menu" of scientific reports.

After this successful start, we would like to thank the authors, the reviewers, the entire production team, and all those who have supported CAPMH during its first year of life. The road ahead looks both promising and challenging. The two main aims for the next two years are to achieve registration in Medline and at Thompson Reuters (in order to receive an impact factor as early as possible). In addition to regular submissions, there will be special sections with invited authors devoted to specific themes of high relevance to child and adolescent mental health. A series of articles on regulatory and ethical issues of child and adolescent psychopharmacological research in the U.S. and Europe is being prepared, and another focused on psychotherapy of posttraumatic stress disorders is under consideration. We welcome suggestions and nominations of high interest topics from the editorial board and all readers. We also have a special interest in case reports with reference to cultural backgrounds and respective mental health systems and would like to encourage relevant submissions from all countries of the world.

We remain aware that one of the challenges for authors is the publication cost which is required in order preserve the open-access nature of the Journal and its complete independence from commercial entities. Still, this "cost of freedom" may be a barrier and discourage submissions. We are examining possible ways of reducing this burden as much as it can be possibly achieved while preserving the open-access, independent characteristics of CAPMH.

We would like to invite all clinicians and researchers in the field, of mental health to contribute to the further development of CAPMH!

## References

[B1] Basker M, Moses PD, Russell S, Russell PS (2007). The psychometric properties of Beck Depression Inventory for adolescent depression in a primary-care paediatric setting in India. Child Adolesc Psychiatry Ment Health.

[B2] Ginicola MM (2007). Children's unique experience of depression: Using a developmental approach to predict variation in symptomatology. Child Adolesc Psychiatry Ment Health.

[B3] Hamerlynck SM, Cohen-Kettenis PT, Vermeiren R, Jansen LM, Bezemer PD, Doreleijers TA (2007). Sexual risk behavior and pregnancy in detained adolescent females: a study in Dutch detention centers. Child Adolesc Psychiatry Ment Health.

